# Exploring the Complexity of Cutaneous Squamous CellCarcinoma Microenvironment: Focus on Immune Cell Roles by Novel 3D In Vitro Models

**DOI:** 10.3390/life15081170

**Published:** 2025-07-23

**Authors:** Marika Quadri, Marco Iuliano, Paolo Rosa, Giorgio Mangino, Elisabetta Palazzo

**Affiliations:** 1 DermoLab, Department of Surgical, Medical, Dental and Morphological Sciences, University of Modena and Reggio Emilia, 41125 Modena, Italy; marika.quadri@unimore.it; 2 Department of Medico-Surgical Sciences and Biotechnologies, Sapienza University of Rome-Polo Pontino, 04100 Latina, Italy; marco.iuliano@uniroma1.it (M.I.); p.rosa@uniroma1.it (P.R.); giorgio.mangino@uniroma1.it (G.M.); 3 Instituto Chirurgico Ortopedico Traumatologico (ICOT), 04100 Latina, Italy

**Keywords:** non-melanoma skin cancer, cutaneous squamous cell carcinoma, 3D models, spheroids, organoids, tumor-on-chip, tumor microenvironment, tumor-associated macrophages, immune cells, immune evasion, immunotherapy

## Abstract

Non-melanoma skin cancer (NMSC), comprising basal cell carcinoma (BCC) and cutaneous squamous cell carcinoma (cSCC), represents the most common type of cancer worldwide, particularly among Caucasians. While BCC is locally invasive with minimal metastatic potential, cSCC is a highly aggressive tumor with a significant potential for metastasis, particularly in elderly populations. Tumor development and progression and the metastasis of cSCC are influenced by a complex interplay between tumor cells and the tumor microenvironment. Recent research highlights the importance of various immune cell subsets, including T cells, tumor-associated macrophages (TAMs), and dendritic cells, in influencing tumor progression, immune evasion, and treatment resistance. This review outlines key regulatory mechanisms in the immune tumor microenvironment (TME) of cSCC and explores the role of cytokines, immune checkpoints, and stromal interactions. We further discuss the relevance of three-dimensional (3D) in vitro models such as spheroids, organoids, and tumor-on-chip systems as tools to mimic immune–tumor interactions with higher physiological relevance, such as macrophage activation and polarization against cSCC cells. Globally, 3D models offer new opportunities for immunotherapy screening and mechanistic studies. Understanding the immune landscape in cSCC through advanced modeling techniques holds strong clinical potential for improving diagnostic and therapeutic strategies.

## 1. Introduction

Non-melanoma skin cancers (NMSCs) represent a heterogeneous group of malignancies, comprising basal cell carcinoma (BCC) and cutaneous squamous cell carcinoma (cSCC), which are the most common types of skin cancer [1]. cSCC typically displays a spectrum of progressively advanced malignancies, ranging from the precursor actinic keratosis (AK), a rough, scaly, or crusty patch precancerous lesion, to in situ, invasive, and metastatic cSCC [2]. The incidence of NMSCs has been rising over the past decades, likely due to increased sun exposure and environmental factors [3]. The exposure to ultraviolet radiation (UV), especially UVA and UVB, which causes DNA damage, is the most significant. Within the environmental exposures, polycyclic aromatic hydrocarbons, nitrosamines, and ionizing radiation also contribute. However, demographic factors, such as fair skin, male, and older age, immunosuppressive conditions, as a consequence of drugs, or other pathological conditions, pre-existing lesions, such as chronic wounds, or genetic syndromes, might be important as risk factors as well [4,5]. BCC mainly presents localized tumors and rarely becomes aggressive and metastatic.

On the other hand, cSCC is more aggressive with significant potential for metastasis, particularly in elderly populations [6,7]. The current therapeutic strategies include surgical excision, but for the most aggressive cancers, it may consist of chemotherapy, targeted therapy, and immunotherapy. However, tumor resistance often leads to treatment failure in a significant number of patients [8,9].

Tumor heterogeneity represents a significant challenge to the development of an effective therapeutic approach [10,11,12]. Patients with the same tumor subtype could present a different tumor phenotype, which may evolve in completely different ways [13]. The mutational events driving carcinogenesis are known to be highly heterogeneous, especially under the influence of UV radiation. This genomic diversity has direct consequences on neoantigen burden, immune editing, and immune surveillance, which in turn shape the composition and function of the tumor microenvironment (TME) [14,15]. The TME, consisting of a complex mixture of cells, including fibroblasts, immune cells, and endothelial cells, immersed in the extracellular matrix (ECM), is important in determining tumor heterogeneity. Furthermore, epigenetic modifications such as DNA methylation, histone modifications, and non-coding RNAs have been shown to regulate the expression of cytokines, immune checkpoint ligands, and antigen processing machinery. These alterations can either promote immune evasion or enhance immune recognition, depending on the context [16].

The complex crosstalk between tumor cells and the components of the TME is responsible for stimulating and promoting an immunosuppressive state, allowing tumor growth, survival, immune evasion, metastasis, and resistance to therapy [17,18,19]. In contrast, normal keratinocytes maintain a cytokine milieu that supports tissue homeostasis and barrier function without promoting chronic inflammation or immune suppression [20]. Specifically, surrounding non-immune cells can sometimes assume some features of immune cells by partially directing or just influencing the immune response, such as cancer-associated fibroblasts (CAFs). Induced by chronic UV exposure and signaling from tumoral cells [21,22], they showed a unique inflammatory gene expression profile [23], specifically associated with the promotion of a more inflammatory state, enacting NMSCs’ invasive properties and angiogenesis [24,25]. Moreover, CAFs in the NMSCs TME secrete various chemokines, cytokines, and ECM components that downregulate the host’s anti-tumor response [22,23,26,27]. This phenomenon is still not completely understood but may resemble what occurs in BCC, where CAFs release factors suppressing anti-tumor immunity after activation through UV radiation and tumor-derived signals [23,28].

Immunosuppression is closely linked to an elevated risk of developing keratinocyte cancer, particularly for cSCC [29]. UV radiation, one of the main risk factors for NMSCs, is known to induce more regulatory T cells than effector T cells in the skin, leading to an immunosuppressive TME that is closely correlated with the development of cutaneous malignancies [30]. Additionally, increased infiltration of specific inflammatory cells, i.e., neutrophils, macrophages, and T lymphocytes, is associated with aggressive cSCC and metastasis [31,32,33]. In this context, tumor-associated macrophages (TAMs) are critical players in TME and are involved in all stages of carcinogenesis [34]. TAMs exhibit a range of functions depending on their polarization into M1 or M2 phenotypes, having an anti- or pro-tumoral effect, respectively, as specifically described below [34,35,36].

Altogether, these findings highlighted the importance of studying TME in NMSCs, especially the interplay between tumor cells and infiltrating immune cells. Therefore, understanding the role of TME is considered a key factor in cancer research, and its targeting might help to enhance the overall effectiveness of cancer treatments.

Traditional research methods have often relied on 2D cell cultures, animal models, and clinical trials. However, these methods have limitations when it comes to replicating the complex in vivo environment of human skin tumors. In the last few years, 3D models have emerged as more accurate platforms for studying the pathophysiology, progression, and treatment of NMSC [37]. Recent advancements in 3D in vitro models have provided a more accurate representation of the complex interactions between epithelial cancer cells and immune cells within the TME, offering insights into the mechanisms of cancer progression and immune evasion [38]. Interestingly, 3D in vitro models have become crucial tools for understanding the role of macrophages in tumor progression. These models help elucidate the molecular mechanisms driving immune evasion and tumor metastasis and hold the potential for developing more effective therapeutic strategies targeting macrophage-tumor interaction, which could significantly improve the treatment outcomes for NMSCs patients. Recently, the development of 3D culture systems by using patient-derived primary cells has revolutionized the impact on cancer studies. These systems have the unique capability to preserve genotypic and phenotypic traits of the original tumor and individual patient characteristics, enabling molecular characterization, drug screening, and the development of personalized approaches. However, the existing 3D culture fails to fully reproduce the complex diversity of TME composition and complex interplay with the entire human body, but they are promising, and, in the future, increasingly sophisticated models will be developed to fill the gap between in vitro culture and animal models.

In this review, we will highlight the importance of TME, particularly the immune cell roles, in cSCC pathogenesis and progression, and will review the most relevant findings about how the interaction between tumor cells and TME generated by using 3D models could apport significant contributions to the development of novel therapeutic approaches for cSCC.

## 2. Immune Cells in cSCC

**Figure 1:**
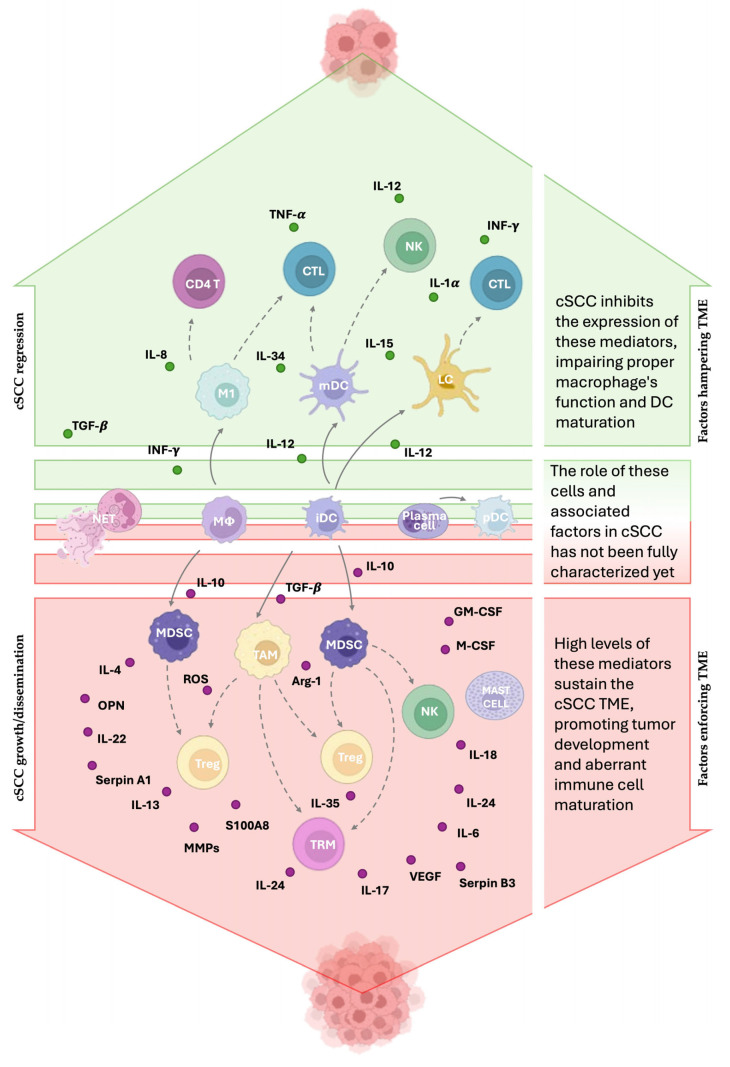
Immune players in cSCC. Immune cells can exert either anti-tumoral or pro-tumoral effects depending on their subtype, localization, and functional state. The TME modifies immune cell behavior to promote cSCC growth. M1 macrophages, Langerhans cells (LCs), and myeloid dendritic cells (mDCs) are hampered by the TME because they contribute to antitumor immunity by enhancing T cell and natural killer (NK) cell activity. Plasmacytoid dendritic cells (pDCs) may play a protective role, while neutrophil extracellular traps (NETs) could favor tumor development, although the exact mechanism is still undefined. Tumor-associated macrophages (TAMs), regulatory T cells (Tregs), and myeloid-derived suppressor cells (MDSCs) support tumor progression through immunosuppressive mechanisms. NK cells and tissue-resident memory T cells (TRMs) can also promote tumor growth through the production of IL-18 and IL-17 cytokines, respectively. The landscape of mediators is difficult to define because their effects vary depending on concentration, duration, and target cells. Mediators marked in green are downregulated in cSCC due to their antitumoral activity, whereas those marked in red are overexpressed because of their protumoral function. The solid arrows indicate how the cells differ from one another. The dashed arrows indicate that the starting cell affects the response of the target cell.

**Figure 2:**
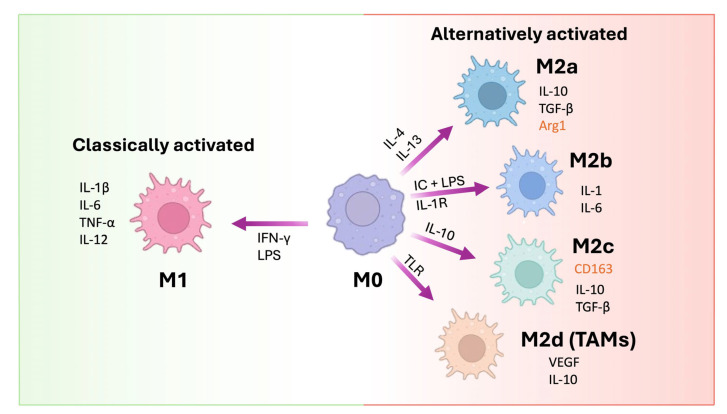
Macrophage polarization pathways. M0 macrophages can differentiate into classically activated M1 macrophages upon stimulation with IFN-γ and lipopolysaccharide (LPS), leading to the production of pro-inflammatory cytokines (IL-1β, IL-6, TNF-α, IL-12). Alternatively activated macrophages (M2) are subdivided into M2a (induced by IL-4/IL-13), M2b (induced by immune complexes + LPS or IL-1R activation), M2c (stimulated by IL-10), and M2d or tumor-associated macrophages (TAMs, induced via TLRs). Each M2 subtype exhibits distinct cytokine profiles and expression markers (marked in red), contributing to tissue repair, immunoregulation, or tumor progression. Arrows indicate the cytokines or signals responsible for each polarization pathway (modified from Gao et al., 2020 [44]).

Immune cells play the most significant role among the non-tumor cells in the TME, having the ability to both limit and promote tumor growth by reciprocal interaction through their molecular and vesicular products [39,40,41,42]. A broader analysis of over 70,000 patients with different cancers supports that generally favorable outcomes are linked to infiltration by CD8^+^ T cells, T helper (Th)1 cells, B cells, natural killer (NK) cells, dendritic cells (DCs), and classical activated macrophages (M1), whereas poor prognosis correlates with regulatory T cells (Tregs), alternative activated macrophages (M2), Th2/Th17 cells, and granulocytes [43]. The overview of the cSCC-immune cell interplay is schematically represented in Figure 1. A detailed analysis of macrophages and their role in cSCC is given in Section 3 and Figure 2.

### 2.1. Antigen-Presenting Cells

Antigen-presenting cells (APCs) include a heterogeneous group of immune cells whose main role is to present antigens to other immune cells to properly produce a correct immune response (i.e., dendritic cells, macrophages). Aberrations in the differentiation of APCs produce immune cells that cannot lead to a correct immune response (i.e., myeloid-derived suppressor cells, tumor-associated macrophages).

In the context of cSCC, myeloid-derived suppressor cells (MDSCs), which are aberrantly activated monocytes or neutrophils, exhibit immunosuppressive functions through the production of arginase, nitric oxide (NO), and reactive oxygen species (ROS). Specifically, NO downregulates E-selectin and impairs T cell entry, thereby limiting T cell infiltration and enabling immune evasion [45,46,47]. The recruitment of MDSCs into cSCC TME could be associated with the overexpression of α6β4 integrin in the upper epidermis, as demonstrated in Invα6β4 transgenic mice. Tumor promoter exposure in these mice leads to elevated levels of C-X-C motif chemokine ligand 5 (CXCL5) and macrophage colony-stimulating factor (M-CSF), promoting the accumulation of CD200R^+^ MDSCs and forkhead box P3 (FoxP3^+^) regulatory T cells while reducing activated CD4^+^ T cells in the skin [17].

In cSCC, there is an increased presence, density, and staining intensity of macrophages compared to normal skin. TAMs do not clearly correspond to classical M1 (pro-inflammatory) or M2 (anti-inflammatory) macrophages, as their functions, secreted factors, and receptors promoting tumor growth are deregulated [48,49]. TAMs help to create an immunosuppressive environment by impairing T cell activity and promoting regulatory T cells and MDSCs. cSCC exhibits higher macrophage levels than normal skin, with TAMs localized inside and around the tumor [49]. A deep explanation about the role of macrophages in cSCC is discussed in the next section.

In humans, cutaneous DCs include Langerhans cells (LCs), myeloid DCs (mDCs), and plasmacytoid DCs (pDCs) [50]. In early cSCC immune response, LCs are crucial APCs, enhancing T cell and NK-cell activity. However, they were found functionally impaired and reduced in cSCC lesions [51,52,53]. mDCs also decreased in cSCC, leading to reduced T cell activation and interferon-gamma (IFN-g) production [53]. Although pDCs are abundant in cSCC, their anti-tumor role via IFN-a remains unclear [51]. They produce IFN-α, possibly supporting anti-tumor immunity [50,54].

Prednisone and Azathioprine are two of the most commonly used immunosuppressive drugs during organ transplantation. They significantly raise the risk of cSCC [55], implying that tumor immune microenvironment alterations may drive cSCC aggressiveness in immunosuppressed individuals. Systemically immunosuppressed patients had fewer CD11c^+^ DCs in tumor centers and developed cSCC at younger ages than immune-competent individuals [56].

### 2.2. T Cells

In cSCC, although T cells infiltrate the tumor, they fail to eliminate it due to increased regulatory T cells (Tregs), which suppress anti-tumor responses [57]. CD3^+^ and CD8^+^ T cells mainly localize to the peritumoral region, while CD4^+^ T cells are found within the tumor. FOXP3^+^ CD4^+^ Tregs, lacking typical skin-resident markers, are elevated and may contribute to immune evasion, metastasis, and display central memory T cell traits [58,59].

CD8^+^ Tregs have also been identified in cSCC [60]. CD8^+^ tissue-resident memory T cells (TRMs) produce interferon-(IFN)-γ, tumor necrosis factor-(TNF)-α, and IL-2, indicating activation potential, but also secrete IL-10, an immunosuppressive cytokine in cSCC. CD8^+^CD103^+^ TRMs are more frequent in cSCC than in non-lesional skin. This scenario favors metastasis [61]. FOXP3^+^ Tregs are more prevalent in cSCC than in normal skin, facilitating immune suppression [59,62]. CD8^+^ Tregs in cSCC are more suppressive than CD4^+^ Tregs [60]. In melanoma, a high percentage of CD39^+^ TRMs is associated with better recurrence-free survival, and TRM-deficient mice are more susceptible to melanoma [63]; however, TRMs are increased in progressive cSCC [64], indicating tumor-specific differences in TRM roles. The study found spatial immune heterogeneity in cSCC lesions: CD8^+^ T cells, co-expressing both activation (Tbet, OX40) and suppression markers (FoxP3, M2-like macrophages), dominated tumor centers, while tumor margins had a more inflammatory Th1/IL-17 profile with Tbet^+^ cells, granulocytes, and DCs. Non-melanoma skin cancers, including cSCC and BCC, show elevated Th1 and Th17 cells compared to normal skin, increased IFN-γ-producing CD8^+^ T cells, and decreased γδ T cells [42,65]. Peritumoral areas of cSCC contain more CD3^+^ and CD8^+^ T cells than normal skin, whereas tumor centers have fewer. 

### 2.3. Other Immune Cells

Neutrophils are the first responders to tissue damage. They fight infections by phagocytosing pathogens, releasing antimicrobial proteins and proteases, and forming neutrophil extracellular traps (NETs). NET is a DNA-based trap released by neutrophils that captures and kills pathogens using attached proteases and antimicrobial molecules, enhancing pathogen clearance in the bloodstream. Moreover, the release of NETs and their homeostatic role could be exploited by cancer [66]. For example, NETs are linked to larger tumor size in melanoma [67], but their impact on NMSCs is still uncertain.

NK cells favor strong, tumor-specific CD8^+^ T cell responses. This occurs through perforin-dependent selection of a more immunogenic subset of DCs capable of activating cytotoxic T cells within TME [43]. In vitro studies demonstrated that treatment with NK led to a dose-dependent decrease in spheroid growth and Matrigel invasion of SCC-13 and HaCaT cells, suggesting that NK cell therapy inhibits cSCC cell growth [68]. In vivo, NK cells help suppress cSCC development by directly targeting tumor cells and interacting with CLEC2A^+^ fibroblasts [69]. However, within the TME, NK cells and the Innate Lymphoid Cells (ILC1s) can upregulate inhibitory receptors, such as CTLA-4 and PD-1, favoring the tumor development [69].

Among immune cell types notably associated with cSCC, there are resting mast cells and activated mast cells. Mast cells are prevalently distributed in the connective tissue, where they principally act as sentinels and exploit a plethora of functions. Among the others, the deregulated actions of mast cells contribute to angiogenesis, ECM remodeling, and immune modulation, all supporting tumor progression and metastasis [70].

## 3. Focus on the Role of Macrophages in cSCC

cSCC is closely influenced by the interaction with the immune system, which can both protect against tumor onset and promote disease progression through immune evasion mechanisms [71]. Due to their innate plasticity and the presence of tissue resident populations, macrophages represent the ideal candidate for transformed cells to interplay with and to hijack immune responses, thus improving tumor settlement, growth, and evading or dampening protective immune responses [36].

Macrophages, indeed, represent one of the first responder populations during both an external and an “altered” self-insult, as they express specific receptors able to recognize pathogen-associated molecular patterns, i.e., PAMPs [72]. The trigger of these PAMPs by bacterial, viral, or self-derived products leads to the activation of a transcriptional program driven by specific transcriptional factors as NF-κB, and interferon regulatory factors, i.e., IRFs, eventually culminating in the synthesis and release of inflammatory cytokines as IL-1β, IL-6, IL-12, and TNF-α, the production of reactive oxygen and nitrogen species, and the increase in their antigen-presenting capabilities. Macrophages activated by such microbial stimuli are referred to as “classically activated” macrophages or, by analogy to the type 1 T helper lymphocytes subset, as M1 macrophages [73]. Once the insult is removed, macrophages are called upon to manage the shutdown of the immune response and to repair the tissue damage caused by both the pathogen and the immune response.

The plasticity of these cells allows macrophages to shift to “alternative” or M2 subsets able to dampen immune response through the release of IL-10 and TGF-β, i.e., M2a macrophages, to contribute to tissue repair through the release of pro-fibrotic factors such as TGF-β, insulin-like growth factor (IGF), and fibronectin, i.e., M2c macrophages, and to promote neo-angiogenesis through the release of vascular endothelial growth factor (VEGF), i.e., M2d macrophages (Figure 2) [44].

Since tumors have characteristics that make them somewhat comparable to cells infected by viruses and/or intracellular bacteria, and they take advantage of their growth of the immunomodulatory and neoangiogenic functions, it is not surprising that tumor cells evolve to create an M2-prone TME. Nevertheless, the so-called “tumor-associated macrophages” or TAMs could display both M1 and M2 phenotypes, also according to the stage of tumor development, as well as to the immunomodulatory capabilities of tumor cells. From this point of view, cSCC behaves like many other types of solid tumors, and many studies have been performed to evaluate the immunomodulatory functions on TAMs.

These studies take advantage of essentially three experimental models: cutaneous biopsies obtained from SCC patients, mice injected with SCC tumor cells or irradiated by UV, and in vitro models using SCC cell lines in co-culture with human macrophages. Even if, as reported below, the majority of the results obtained using these experimental models indicate a neat propensity of cSCC to set a M2-prone TME, some reports indicate the presence or the recruitment of M1-like subpopulations. In patients affected by BCC, a more indolent and less aggressive form of skin cancer, tumor-infiltrating lymphocytes (TILs) express both type 1, i.e., IL-2 and IFNγ, and type 2, i.e., IL-4 and IL-10 cytokines, with the former mainly produced by TILs and the latter by adherent, TIL-depleted cells [74]. Samples obtained from cutaneous nodular stage 1 cSCC patients reveal that both CD163^+^ and CD68^+^ macrophages were enriched in lesional compared to non-lesional skin, and that some of them express pSTAT6, a marker typically associated with type 2 polarization, whereas others express pSTAT1 and IFNγR1, typically associated with type 1 polarization. Further, when looking at the M1/M2-associated gene sets, lesional skin appears to overexpress the M1 gene set compared to non-lesional skin [49].

These observations were also confirmed in cSCC biopsies collected from patients who received a solid organ transplant (i.e., SOTRs), a condition that leads to a 100-fold higher risk of development of cSCC. In this case, no quantitative differences in the number of intra-tumoral and peritumoral macrophages were observed, nor a difference in the number of macrophages in lesional versus non-lesional skin. On the other hand, the intra-tumoral infiltration of macrophages in cSSC in situ was lower in SOTRs compared to non-transplanted subjects, suggesting that in the former group, early macrophage recruitment is impaired. In addition, in SOTRs M2 TAMs are underrepresented compared to non-transplanted individuals [75]. Using a transgenic mouse model expressing HPV8-derived E6 in keratinocytes, it has been reported that in UV-induced cSCC, Ly6C^hi^ cells accumulated in the lesions and that these cells are mostly M1-like macrophages derived from circulating monocytes recruited to the tumor, as the specific depletion of these monocytes made the mice resistant to UV-induced SCC [76].

One of the most striking characteristics that most of these observations share, is that they were performed in early-stage SCC or in non-invasive tumors as BCC. It is, then, tempting to speculate that in less aggressive tumor and early in the settlement of cSCC, macrophages can recognize tumor cells as a danger, thus mounting a typical M1 protective response. This response, in turn, selects tumor cell variants that are prone to manipulate the TME, creating the conditions for the shift from M1 to M2 macrophages, which most of the studies on SCC reported as the main TAM subset [77]. Since the mid-nineties, indeed, it has been reported that in UV-irradiated skins, IL-10 is overexpressed and that CD11b^+^ infiltrating macrophages are the main producers of IL-10, suggesting that these experimental conditions can settle a M2-favorable TME [78,79]. These observations parallel those obtained in skin biopsies collected from BCC and cSCC patients demonstrating that BBC express higher levels of type 2 cytokines as IL-4 and IL-10 compared to normal skin and that the cellular source of these cytokines are cells depleted on non-adherent lymphocytes, probably a mix of macrophages and tumor cells adhered to cell plates. Even more strikingly, in the case of cSCC, all the tested patients expressed high levels of IL-10 [78].

The presence into the tumor of IL-10 indicated the presence of the M2a and/or M2c subset even if later on, other authors reported also that the presence of high levels of VEGF-C in SCC lesions is associated with the presence of CD163^+^ and/or CD68^+^ peritumoral macrophages strongly suggesting that TAMs may also display a M2d phenotype [18]. Depending on the phenotypic characterization of TAMs by flow cytometry, it has been reported that SCC have a higher TAM density than BCC and that these infiltrating macrophages express higher levels of arginase-1 and MMP-9 (both typically M2 markers) as well as CD40 and CD127 (considered M1 markers), in SCC compared to BCC. These observations were confirmed using the THP-1 monocytic cell line treated with supernatants collected from both SCC and BCC [32]. Further, when stratified SCC patients according to α3 Laminin expression it has been demonstrated that lower α3 Laminin expression confers tumor cells a more invasive behavior and that these cells release higher amount of CCL-2/MCP-1 and IL-13 [80]. Using a xenograft SCID mice model these authors also demonstrated that CCL-2/MCP-1 is responsible for the monocyte recruitment into the tumor whereas IL-13 polarize these cells into M2 subset characterized by high expression of arginase-1 and low expression of inducible nitric oxide synthase (iNOS) [80].

SCC cells are able to manipulate the TME not only through the release of M2 polarizing cytokines but also through bioactive molecules as circular RNAs (circRNAs). Indeed, it has been reported that in cSCC tissues, cells circ_TNFRSF21 is overexpressed and, when SCC cells are cocultured with THP-1 cells, they induce on this monocytic cell line a M2 phenotype which is reverted when SCC cells are circ_TNFRSF21 silenced [81]. High expression levels of circ_TNFRSF21 were also found in SCC lesions and correlated with CD163 expression. Furthermore, supernatants collected from SCC cell lines induced THP-1 polarization into M2 macrophages, which was impaired in circ_TNFRSF21-silenced THP-1. Most importantly, supernatants collected from M2 macrophages induce tube formation in HUVEC cells and, again, this effect is impaired when circ_TNFRSF21 is silenced, indicating that in this context, M2-like TAMs could play a pivotal pro-angiogenic role [81]. Interestingly enough, also the opposite, i.e., the ability of M2 macrophages to increase the survival of SCC tumor cells via circRNAs, is true. In this case, it has been demonstrated that M2 TAMs convey circ_0088494 to SCC tumor cells through the release of small extracellular vesicles (EVs) able to transfer circ_00884 into tumor cells, thereby impairing ferroptosis, an iron-dependent type of cell death, and promoting tumor cell survival [82]. Small EVs, formerly defined as exosomes, are also used by tumor cells to push M2 polarization. Indeed, in a mouse xenograft model, it has been demonstrated that, when THP-1 were co-injected into mice with A431 SCC cells overexpressing the SNF2-like family member CHD1L, a protein which play a role into DNA repair, the maintenance of chromosomal integrity and transcriptional control, macrophages polarize into M2 cells, whereas they become M1 when co-injected with A431 cell knocked out for CHD1L [74].

### Macrophage Targeting

As it has been clearly demonstrated that the presence of M2-like TAMs is associated with a poor prognosis in SCC patients [81]. It is not surprising that interventions leading to M2 macrophages inhibition and/or to a shift into M1 subset are nowadays considered as a therapeutic approach standing alone or in combination with other immunomodulatory therapies. In fact, it has been reported that alkannin, a derivative compound from the roots of *L. erythrorhizon*, is able to induce both in vivo and in vitro, M1 polarization and cSCC cell apoptosis through the activation of PTEN pathway [83]. Using a highly aggressive derivative of PDV murine cSCC cell line (i.e., PDVC57 cells), it has been observed that mice injected with the PDVC57 cells develop tumors in all animals and that these SCC are characterized by a highly M2 TAMs infiltration and a higher arginase-1 enzymatic activity. Using the arginase-1 inhibitor Nω-hydroxy-nor-arginine (nor-NOHA) alone or in combination with the PD-1 inhibitor nivolumab, a significant reduction in arginase-1 activity and tumor mass was recorded [84]. Topical administration of 5-aminolevulinic acid in association with photodynamic therapy (PDT) in a murine cSCC model and patients allows the increase of CCL8/MCP-2 expression, the recruitment of macrophages with an M1 phenotype, and the regression of tumor growth in mice, especially when co-administered with exogenous CCL8/MCP-2 [85]. A similar mechanism has also been reported in the case of methionine enkephalin (MENK), also known as opioid growth factor (OGF), which is able to inhibit cSCC cell growth in vivo in a xenograft nude mice model. In addition, in OGF-treated mice, TAMs express the M1-associated marker CD86+ to a greater extent than in the control group, where TAMs predominantly express the M2-associated marker CD206 [86].

## 4. Immune TME Regulators

Several factors can influence the ability of the immune cells to regulate the cSCC microenvironment, both directly by cytokine/chemokine release and indirectly through pharmacological regulation. A summary of the main immune TME regulators in cSCC is in Table 1. Immunosuppressive agents contribute to skin carcinogenesis by hindering DNA repair, weakening the immune system’s ability to eliminate malignant cells, and increasing the expression of cytokines that support tumor growth [87].

### 4.1. Immune Cells Receptors

Among the immunoregulatory factors that influence the composition and behavior of the TME in cSCC, CD200 is a protein that plays a significant role in modulating immune responses and promoting an immunosuppressive milieu. TAMs may overexpress CD200, which inhibits pro-inflammatory maturation of macrophages or DCs [18,88], and produce matrix metalloproteinases (MMPs) that aid invasion and lymphangiogenesis via vascular-endothelial growth factor-(VEGF)-C expression [89]. CD200 acts by binding to CD200R on NK cells, T cells, MDSCs, and TAMs [107]. The CD200-CD200R axis promotes metastasis in cSCC by activating Cathepsin K (Ctsk), a collagen proteinase more effective at degrading type I collagen than MMPs, enhancing cSCC invasion [88]. CD200 is also highly expressed by blood vessel endothelial cells in cSCC, contributing to immunosuppression in the TME [89]. Another oncogenic contributor to the immunosuppressive TME is SHC binding and spindle-associated 1 (SHCBP1), a signaling regulator associated with tumor growth. High SHCBP1 expression correlates with lower CD8^+^ T cell infiltration and higher levels of M2-TAMs, MDSCs, CAFs, and Tregs, along with upregulation of immune checkpoint molecules, which help tumors evade immune detection and reduce T cell-mediated killing [91].

Programmed death ligands (PD-L1 and -L2), by binding PD-1, suppress the proliferation of PD-1-positive cells, reduce their cytokine production, and promote apoptosis. They are crucial for regulating immune responses and maintaining self-tolerance by modulating T cell activity. PD-1 promotes the apoptosis of antigen-specific T cells while preventing the apoptosis of regulatory T cells [92,108]. PD-L1 expression is significantly elevated in sun-exposed skin of cSCC patients [109]. Moreover, PD-L1 and -L2 are present on tumor-infiltrating DCs, especially CD1a^+^ subsets, potentially suppressing anti-tumor immune responses and aiding immune evasion in cSCC [93].

Some other immunosuppressors that could be important in cSCC development are cytotoxic T lymphocyte antigen 4 (CTLA-4), which increases after T cell activation to suppress immune responses, and lymphocyte-activation gene 3 (LAG-3), which acts similarly to PD-1 and CTLA-4 in downregulating T cell activity [41].

### 4.2. Cytokines and Chemokines

Cytokines and chemokines play key roles in tumor cell proliferation and invasion, but their effects vary depending on the tumor stage due to complex interactions [110]. IL-6 initiates a cytokine network that promotes malignant growth and tumor cell migration [96]. Higher IFN-γ levels are linked to advanced cancer stages, especially when analyzed alongside TGF-β, with mild correlations also seen between IFN-γ and granulocyte-macrophage colony-stimulating factor (GM-CSF) or GM-CSF and TGF-β, suggesting interdependent expression [110]. IL-24 overexpression in invasive cSCC increases MMP-7, enhancing proliferation and migration [97]. G-CSF and GM-CSF are constantly expressed in SCCs, supporting growth and metastasis by promoting proliferation and angiogenesis [111]. The role of TGF-β1 strongly depends on the stage of cSCC: while in the early stage, it acts as a tumor suppressor, it contributes to tumor progression in later cSCC stages, by enhancing tumor heterogeneity and treatment resistance via cancer stem cell pathways, increasing invasiveness by promoting epithelial to mesenchymal transition, and suppressing anti-tumor immunity. Such behavior depends on the different receptors that TGF-β1 can interact with from early to late stage: TGF-βRI and TGF-β1RII, respectively [98]. IL-22, elevated in peritumoral cSCC areas, promotes proliferation and reduces IFN-γ production by Th1 cells [99]. A comparison of keratoacanthoma (KA) and cSCC revealed distinct TMEs: IL-27 was increased only in KA, while MMP-9 was found only in cSCC [101]. IL-27, produced by activated APCs, promotes naïve CD4^+^ T cell proliferation and T-bet expression, fostering a Th1-like TME. In contrast, MMP-9, produced by M2-TAMs, is involved in invasion and metastasis and may be linked to poor prognosis [101].

### 4.3. Angiogenic Factors

In cSCC, the formation of new blood and lymphatic vessels (angiogenesis and lympho-angiogenesis) is linked to deeper tumor invasion and poorer differentiation [112]. Moreover, both processes are active, with upregulation of genes like VEGF-C, neuropilin 2 (Nrp-2), and VEGFR-3 [113]. While lymphatic vessels usually support immune responses by transporting cells and antigens to lymph nodes, chronic immune activation can shift the environment to one that promotes tumor growth. cSCCs exhibit elevated VEGFs, increased lymphatic vessel area (LVA), and lymphatic vessel density (LVD), with significantly higher expression of angiogenesis and lympho-angiogenesis-related genes than normal skin [102]. Using a murine multistep chemical carcinogenesis model, researchers studied a soluble VEGF-C/VEGF-D inhibitor and found that both VEGF-C and VEGF-D contribute to an inflammatory tumor microenvironment promoting early tumor progression, while VEGF-D also drives lympho-angiogenesis and late-stage metastasis via VEGFR-3 signaling [114]. Inhibiting VEGF-C and VEGF-D reduced blood vessel density in skin and tumors [113]. In another murine SCC model, VEGF-D influenced the immune microenvironment by reducing Th2 inflammation, increasing CD8^+^ T cells, and decreasing CD4^+^ T cells, macrophages, and mast cells, leading to tumor regression in early stages. Although VEGF-D raised IL-4 levels (an M2 pro-tumoral cytokine), overall IL-4 expression was still low, and its effects were context-dependent [115]. Ultimately, VEGF-D has dual roles: it can suppress early tumor growth by promoting Th1/Th17 immune responses but also enhances lymphatic spread and metastasis through VEGFR-3 signaling [116].

### 4.4. ECM Regulating Factors

The cytokine-like activities of S100 calcium-binding protein (S100)A8 and S100A9 vary depending on the tumor type, and they are rarely found in normal tissues [117]. These proteins are highly expressed in cSCC and play a significant role in tumor development, while their expression is low in AK [103]. Microbial structures and inflammatory cell infiltration stimulate the secretion of proteases like MMPs, which remodel the ECM and basement membrane. This remodeling affects the availability and activity of growth factors, cytokines, and chemokines, promoting inflammation and creating conditions favorable for cSCC development [104]. Chronic UV radiation, immunosuppression, and human papillomavirus (HPV) infection further increase susceptibility to cSCC [118].

MMPs are enzymes able to degrade ECM components and modulate signaling molecules, influencing tumor cell behavior. To fine-tune their actions, MMPs exist in various forms, each with distinct molecular targets, tissue localization, and modes of activation. For instance, MMP-13 promotes invasion at the tumor margins, while MMP-7 activates growth factors to drive proliferation [119]. In cSCC, the most expressed MMPs by tumor cells and stromal cells are MMP-1, MMP-7, MMP-9, MMP-13, and MMP-14. Another type of protease with a function similar to MMPs is serine protease inhibitors (serpins). For example, serpinA1 (α1-antitrypsin) is upregulated in tumor cells, promoting cancer by inhibiting apoptosis, suppressing immune responses, and stimulating proliferation [105]; while serpinB3 (SCCA1) is a marker of advanced cSCC disease stages [120].

The complement system, typically part of immune defense, is also active in cSCC, contributing to tumor progression by promoting inflammation, vascularization, and cancer cell survival. Complement regulatory proteins like CFH and CFI are more strongly expressed in invasive cSCC and modulate protease activity through interactions with molecules like osteopontin (OPN) [121]. OPN exists in two forms: secreted (sOPN) and intracellular (iOPN). sOPN plays a key role in immune responses, cancer progression, and acts as a chemoattractant for immune cells. It also enhances Th1 immune responses, anti-apoptotic effects, and production of immune-stimulating molecules like IFN-γ [122]. iOPN, found in antigen-presenting cells, contributes to immune regulation. OPN plays a significant role in cSCC, as it is found in metastatic SCC and AK, but is absent or low in non-metastatic tumors such as solid BCC [106]. In cSCC, OPN enhances tumor progression and metastasis by promoting cell migration, invasion, and survival via the activation of integrin and CD44-mediated signaling pathways. These interactions result in increased matrix degradation, epithelial–mesenchymal transition (EMT), and a pro-inflammatory tumor microenvironment that facilitates metastatic spread [106].

## 5. 3D Models for the Study of the Tumor Microenvironment in cSCC: Focus on Immunity

Tumors have been demonstrated to be part of a more complex environment consisting not only of tumor cells but also of a variety of other cells, such as immune cells, endothelial cells, and fibroblasts, soluble factors, and ECM components, which are able to influence tumor progression, metastasis, and therapeutic efficacy.

Immunotherapy has been an important advancement in cancer treatment, including cSCC, especially with the introduction of immune checkpoint inhibitors or immune-cell therapy. However, while this treatment supports good initial patient survival, it often fails due to the development of resistance mechanisms [69], highlighting the urgent need to understand the complex network between cancer and TME. Unfortunately, as described above, the interaction between tumors and immune cells is complex. Cancer cells activate various mechanisms to evade immunity, making it essential to understand these processes for the development of new therapeutic strategies. The immune system consists of various cell types that interact with each other, and soluble factors with diverse functions, which can result in either anti-tumor or pro-tumor effects [123,124]. This overview underscores the challenge of replicating these interactions and highlights the importance of understanding this mechanism to develop new molecular targets and specifically design immune cell-based therapies [125].

Several difficulties have been encountered in studying the interplay between tumor cells and TME. The best option would be represented by in vivo models; however, they are expensive to maintain and subject to strict ethical rules [126]. Specifically, murine models of cSCC, including genetically modified mice and UV-induced cSCC [37], have provided valuable insights into the polarization, recruitment, and spatial distribution of tumor-associated macrophages (TAMs) within the tumor microenvironment [39,127]. These models have enabled researchers to study the dynamic transition from pro-inflammatory M1 macrophages to immunosuppressive M2 phenotypes during tumor initiation and progression, as well as the molecular signals—including cytokines, chemokines, and tumor-derived factors—that drive these changes. Murine systems have revealed how TAMs contribute to immune evasion, neoangiogenesis, and tumor invasion, thereby supporting the establishment of a tumor-promoting microenvironment [44,49].

To partially simulate the complex interaction between tumor cells and stromal cells, several co-culture systems were established, where cancer cells and stromal or immune cells were cultured together in a 2D system. Moreover, it is also possible to evaluate the influence of paracrine factors by using conditioned media. A more sophisticated 2D method involves the use of Transwell cell culture, which allows for the cultivation of several cell types in the same environment, sharing the same medium. In this system, cells can communicate through soluble factors. Interestingly, the two cell compartments are divided by the Transwell membrane, also allowing for the evaluation of chemoattractant factors, migratory capacity of cancer cells, and their ability to bypass the ECM and extravasate by pre-coating the top of the membrane with ECM components (i.e., collagen or Matrigel) or endothelial cells and evaluating immune cell recruitment. 

By using these 2D models, Toutfaire and co-workers show that cSCC cell lines highly induce and reinforce the senescence of human diploid fibroblast (HDFs), which is modulated according to the stage of tumorigenesis of the different cSCC cell lines used [128]. As regards to immune system, Quadri et al. were able to demonstrate that CD271 overexpressing cSCC cells increase recruitment and macrophage differentiation [38].

Given their cost-effectiveness, availability, high-throughput, easy replication, and results’ interpretation, 2D cell cultures are still widely used, particularly to obtain preliminary data. However, 2D culture methods are really limited. Monolayer growth changes cells’ original morphology and polarization, potentially disrupting critical signaling pathways and altering responses to external stimuli [129,130]. Additionally, cells in 2D may undergo clonal selection, losing the genetic heterogeneity of the original tumor. Furthermore, these models fail to accurately represent the primitive intrinsic tumor stroma and its three-dimensional architecture [130,131,132,133,134,135,136].

**Figure 3:**
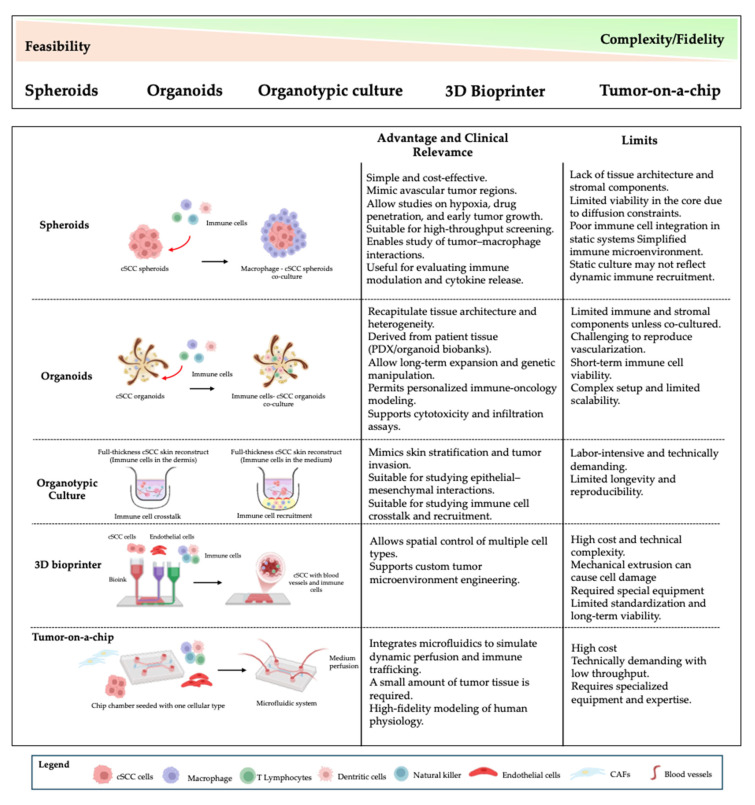
Overview of 3D experimental models used to study cSCC and tumor–immune interactions. A range of 3D culture systems is available to model cSCC, each differing in complexity, biological fidelity, and suitability for immune cell integration. Spheroids and organoids are accessible and reproducible, supporting initial tumor characterization, while more advanced systems like organotypic cultures, 3D bioprinting, and tumor-on-a-chip platforms enable higher architectural and microenvironmental complexity. Co-culture approaches and immune cell incorporation strategies (e.g., macrophages, T cells, dendritic cells) allow investigation of immune–tumor crosstalk. This figure summarizes their feasibility, immune relevance, and experimental potential in modeling immune recruitment and tissue organization.

In the last decade, trying to recreate the situation in vivo, researchers have developed 3D in vitro models with different degrees of complexity, starting from tumor cell aggregation and, successively, gradually integrating components of the TME to finally reconstruct the complete tissue from both structural and immune points of view. Here, we describe 3D in vitro models developed to study the complex crosstalk between tumor cells and TME, focusing on 3D cancer-immune models, which are emerging as patient-relevant in vitro tools for modeling tumor-immune interaction [37]. A schematic representation is given in Figure 3.

### 5.1. Spheroids

Tumor spheroids are one of the most used 3D models in cancer research. They consist of small, spherical clusters of cells that are prevented from adhering through several processes (reviewed in [37,133]) and then aggregate into a 3D structure. This 3D architecture better mimics the complex structure and behavior of tumors in vivo, including gradients of oxygen, availability of nutrients, cell–cell adhesion, and cell–matrix interactions. Spheroids can be generated from tumor cell lines and, surprisingly, with patient-derived cells. Recently, the characterization of patient-derived cSCC spheroids of different stages has been shown. Interestingly, Quadri and co-authors have demonstrated that patient-derived spheroids were able to reproduce the characteristics of the tumor in vivo in terms of gene and marker expressions, differentiation state, and cell proliferation [38], demonstrating that spheroids are an invaluable tool for understanding tumor growth, invasion, and testing drug efficacy.

To recreate the complex interaction between tumor and TME, co-cultural spheroids, also called “tumoroids” or “heterotypic spheroids”, have been developed, incorporating tumor cells and stromal, i.e., fibroblast, endothelial cells, and/or immune cells [137,138]. Surprisingly, primary neoplastic cells could be heterogeneously assembled with infiltrating or resident host non-neoplastic cells (i.e., T and B lymphocytes, natural killer, dendritic cells, monocytes, endothelial cells, CAFs, mesenchymal stromal cells, etc.) alongside niche-specific soluble factors, such as cytokines, growth factors, metabolites, enzymes, miRNAs, and ECM components [139], allowing the complete recreation of the TME.

A 3D spherical model has recently been developed utilizing biodegradable gelatin-based microcarriers in a spinner flask bioreactor, co-culturing pancreatic cancer cells with CAFs. In this system, spheroids reach 400 μm in size, reproducing oxygen and nutrient gradients like in vivo conditions, making them a suitable model for investigating the impact of hypoxia on cancer progression, blood vessel formation, and drug effectiveness [140,141].

As a concern for cSCC, Siljamaki et al. established 3D spheroid co-cultures using primary skin fibroblasts and HaCaT/ras-HaCaT human keratinocytes, demonstrating the influence of fibroblasts on cSCC progression and invasion [142]. Dayal and co-workers also set up a co-culture model with fibroblasts with recessive dystrophic epidermolysis bullosa (RDEB) cSCC keratinocytes (SCCRDEB3). They found that fibroblasts overexpressing type VII collagen increased structural organization when co-cultured with SCCRDEB3 respect to control fibroblasts [143].

Investigating the interaction between tumor cells and the immune system, heterotypic spheroids have been recreated, which is useful for studying tumor-immune system interactions and testing immunotherapeutic agents. Recently, a heterotypic spheroid co-culture model of human colorectal cancer with immune cells was established, demonstrating that allogeneic T and NK cells infiltrated cell line-derived spheroids, leading to immune-mediated cancer cell killing and destruction of the 3D structure. Moreover, the authors were able to develop autologous spheroidal models with patient-derived cells and patients’ tumor-infiltrating lymphocytes, which have been revealed to be an excellent clinical tool to study the response to immunotherapies [144].

As concern for cSCC, Adhikary et al. evaluated the ability of NK cells derived from healthy adult donors to suppress the cSCC cell cancer phenotype and reduce tumor growth. In particular, they set up an interesting 3D models by seeding human NK cells together with cSCC spheroids, demonstrating that NK cells produced a dose-dependent reduction in SCC-13 and HaCaT spheroid growth and Matrigel invasion by inducing cell apoptosis [68]. On the other hand, Quadri et al. developed a cSCC immune-spheroids model by co-culturing SCC-13 spheroids with the human monocyte cell line THP1. The model showed that CD271-overexpressing cSCC spheroids increased monocyte infiltration into tumor spheroids [38].

Interestingly, it has recently developed a high-throughput 3D tumor spheroid microarray, which comprises a 330 micropillar-microwell sandwich platform. In this setup, NK cells are co-cultured with pancreatic (MiaPaCa-2) or breast cancer cell lines (MCF-7 and MDA-MB-231) to accurately replicate the hypoxic TME and investigate NK-cell mediated cytotoxicity in combination with the monoclonal antibodies Trastuzumab and Atezolizumab [145].

Research on 3D spheroid-based models is still growing and may apply to different types of tumors, including cSCC. However, their static nature restricts our understanding of the complexity of tumors and the TME in vivo.

### 5.2. Organoids

Although tumor spheroids better mimic the situation in vivo than 2D monolayer culture, they fail to accurately mimic the intricate biological and clinical characteristics of primary tumor tissues. Research advancements in new in vitro models have resulted in the development of organoids, which are clusters of cells that grow in a defined three-dimensional in vitro environment. In this environment, they self-organize and differentiate into functional cell types, mimicking the structure and function of a natural organ/tumor in vivo [146]. Organoids can be created from embryonic stem cells, induced pluripotent stem cells (iPSCs), or neonatal/adult stem cells, reflecting the development of an organ’s unique structure [147].

In cancer research, organoids have gained significant attention for their ability to preserve the essential structural and functional characteristics of real tumors. They potentially offer a highly predictive in vitro model for guiding clinical decisions [148]. Organoids could be useful for high-throughput drug screening, disease modeling, and clinical translation. They can recreate complex TME by co-culturing with immune cells or other TME-associated cells [149]. Recently, extensive tumor biobanks have been established through 3D cultivation of patient-derived organoids (PDOs), which maintain the genetic and histological diversity of the original tumor tissues [150]. The PDOs culture system has been developed and applied to various types of cancer, including breast, colon, gastric, prostate, pancreatic, and renal carcinoma [151]and would be potentially applicable to the study of cSCC. These “living biobanks” of PDOs capturing the histological and mutational heterogeneity of humans provide a representative collection of well-characterized models for preclinical drug screening and for predicting patient outcomes, as extensively discussed by others [37,152,153]. However, they are costlier and harder to use [154].

Recently, researchers have improved organoids by adding different elements, such as immune cells, CAFs, tumor vasculature, and other biological or chemical components, trying to replicate the TME. However, the incorporation of immune cells into organoids is still ongoing, with few successful studies [155].

Interestingly, Song et al. recently developed a method for generating and biobanking high-fidelity patient-derived glioblastoma organoids for testing personalized therapies and model CAR T cell-based immunotherapy [156]. Moreover, a more complex model has been established, composed of a triple co-culture of mouse gastric tumor organoids, DCs, and CTLs. The authors were able to demonstrate that, in the presence of anti-PD-L1 neutralizing antibody, antigen-stimulated CTLs to kill gastric tumor organoids [157]. Moreover, Ramsay and colleagues co-cultured human colorectal cancer organoids with tumor-infiltrating T lymphocytes (TILs), demonstrating that the exposure to an anti-PD-1 antibody partially restored the antitumor immunity of PD-1-expressing T cells [158].

All these studies suggest that the reconstitution of a functional tumor-immune model may allow the study of tumor–immune and immune–immune cell crosstalk, allowing the development of higher-efficacy immunotherapies. Unfortunately, no studies reported the use of organoids with an immune system for the study of cSCC. However, considering the significant impact of these models and the role of the immune system in cSCC, the reporting of such information would be beneficial for researchers in these fields to develop an immune-organoid cSCC model.

### 5.3. Organotypic Culture or Skin Reconstruct

Organotypic culture models accurately replicate skin architecture by incorporating human fibroblasts and extracellular matrix components (such as Matrigel or collagen) to form the dermal layer, while human keratinocytes are used to construct the epidermis. On the other hand, in tumor skin reconstructs, keratinocytes are combined with tumor cells (such as melanoma, cSCC, or BCC). The protocol includes: (i) dermis construction and contraction on a Transwell insert; (ii) seeding of healthy keratinocytes and tumor cells on the dermal compartment; (iii) growth in immersion conditions; (iv) exposure to air-liquid interface. This crucial step enables the formation of skin cell layers with physiological traits like differentiation and tissue organization, key for studying skin and skin cancer biology [75]. These models are particularly useful for studying tumor progression and invasiveness, their interactions with the surrounding ECM, and the effects of different treatments. They can be derived from both cancer cell lines and patient-derived cells [38,159,160]. Moreover, a spheroid-organotypic model has been developed in which tumor spheroids are embedded within the dermal compartment to study tumor metastasis [37].

The use of cSCC skin reconstructs is widely reported [37]. Quadri et al. established skin reconstructs by using SCC13 cell lines transfected with CD271 viral vector, demonstrating that CD271 overexpression increased cSCC differentiation [38]. On the other hand, Dallaglio and colleagues generated SCC13-derived rapidly adherent cells (RAD cells) and non-rapidly adherent (NRAD cells) to collagen IV skin reconstructs, defining the role of Survivin stem-like cSCC cells [161].

Skin is a complex tissue comprising several cell types in addition to fibroblasts and keratinocytes, such as immune cells, sensory cells, and endothelial cells. During the last years, several organotypic models have been developed to fully recapitulate the complete architecture of healthy and pathological skin, allowing the study of its biology from different points of view [162,163,164]. For instance, Morgner and colleagues established psoriasis and atopic dermatitis-like phenotypes in 3D skin equivalents with a fibroblast-derived matrix [165]. They reproduced the pathological skin condition by adding T cell signaling of psoriasis and atopic dermatitis by adding Th1 and Th2 cytokine stimulation [165].

The concept of a “full thickness skin” was also applied to tumoral-skin reconstructs to recapitulate the tumor-TME interactions in a more physiological context. CAFs, instead of normal fibroblasts, were used in cSCC skin reconstruction to examine their impact on tumor progression and invasion [166,167]. A 3D organotypic skin culture model containing NK cells was recently developed to unveil the important crosstalk between fibroblasts and NK cells in SCC invasion. Thanks to this model, the authors found that CLEC2A has a key role in orchestrating the crosstalk between fibroblasts and NK cells, thereby leading to the control of cSCC invasion [168].

The versatility of these models is significant, as they accurately replicate both the skin and the tumor within its TME. In the context of the immune system, it would be possible to study both the immune cells’ crosstalk and the immune cells’ recruitment process, depending on how the organotypic cultures are reconstructed. For instance, skin tumor reconstruction may be performed by incorporating endothelial cells beneath the dermis, facilitating the formation of a vasculature-like structure, and immune cells within or below the dermal compartment to study immune cells crosstalk and immune cells recruitment, respectively.

This model has a wide range of applications. However, their static nature means that they still have numerous limitations and are unable to fully recreate the situation in vivo.

### 5.4. 3D Bioprinting

3D bioprinting is a more modern approach, which involves the use of computer-aided design technology. This method uses a 3D printer that precisely places cells in a layer-by-layer manner to form complex structures. Thanks to the availability of a wide range of printing materials and manufacturing processes, this technology enables the creation of a more reproducible physiological tissue by adding cells, bioactive molecules, and biomaterials. This method allows the creation of more advanced tumor models with multiple cell types, including immune cells, fibroblasts, and endothelial cells. It enables the creation of highly reproducible, accurate, and stable models with control over cell placement, allowing researchers to create customized TME or study tumors in various stages [169].

Recently, the development of patient-specific bladder mini-organs using 3D bioprinting has been shown. This model maintained the genetic changes of the original tumor and showed that the interaction between tumor and stromal cells is crucial in controlling tumor plasticity [170]. Consistently, Heimrich and colleagues were able to establish a “mini brain” by mixing glioma cells with macrophages, demonstrating the importance of macrophages in inducing cancer proliferation and invasion [171].

Two works report on the development of a 3D bioprinting model for cSCC. In 2020, Browning et al. created a 3D bioprinted cSCC model using a bio-fabricated full-thickness skin. After 4 days of media submersion, A431 cSCC spheroids were introduced. This model reproduced tumor invasion and proved useful for testing chemotherapeutic efficacy and toxicity [172]. In 2024, Kurzyk et al. developed a 3D bioprinting model of human cSCC using HaCaT keratinocytes, primary dermal fibroblasts, and A431 cSCC cells. This model highlights the potential of 3D bioprinting in studying drug responses and the interactions within the TME [173].

3D bioprinting presents broad applicability in the generation of highly precise and reproducible tumor models, effectively recapitulating the structural and biochemical properties of the ECM and TME. Nevertheless, its implementation in high-throughput screening remains limited due to technical complexity, and the use of bioinks and associated fabrication processes may adversely impact cell viability and functional integrity [174].

### 5.5. Tumor-on-a-Chip Model

An emerging example of 3D technology is the multichannel microfluidic perfusion culture system, commonly referred to as an “organ-on-a-chip” or, specifically for cancer, “tumor-on-a-chip”. This platform utilizes microfluidic devices, enabling the dynamic simulation of tissue- and organ-level functions within a controlled microenvironment. The system comprises distinct compartments populated with various cell types, such as endothelial and mesenchymal cells, with or without the inclusion of ECM components. The microfluidics parts replicate the conditions found in living organisms, such as blood flow with nutrients and oxygen transport. By preserving the structural architecture of native tissues, these platforms facilitate the investigation of interactions between cancer cells and the surrounding stromal environment in a more physiological context [175,176].

The advantage of this model also comprises the precise control of the physiological conditions, compatibility with several analytical techniques, and faster experiment timelines [139]. The integration of these models into industrial high-throughput drug screening, where thousands of compounds are simultaneously evaluated, poses the challenge of developing precise, automated analytical systems capable of generating critical data to inform compound optimization [177]. More recently, the development of ‘spheroids-on-a-chip’ platforms has emerged as a promising preclinical approach for investigating tumor angiogenesis, metastatic potential, and therapeutic responses for different cancer types.

In the context of cSCC, skin-on-a-chip models may be more suitable methods as they simulate the physiological environment of the skin. These models could be particularly useful for examining the interaction between cSCC cells and skin-resident immune cells, as well as for testing new treatments or understanding metastasis to other skin areas [37,178,179,180,181,182,183]. Recently, Michielon and colleagues developed a reusable multi-well skin-on-a-chip model incorporating both immune and endothelial cells. This model provided proof of concept for immune cell circulation and activation within the skin environment [179]. Unfortunately, a cSCC-on-a-chip model has not yet been developed. However, the ongoing advancement of these technologies is likely to lead to its creation soon.

## 6. Conclusions

In conclusion, the use of complex three-dimensional in vitro models has significantly advanced our understanding of the mechanisms underlying the progression and aggressiveness of cSCC, particularly highlighting the pivotal role of the immune cells and, namely, of the macrophages within TME. Our comprehensive review of all the recent technologies and applications within cancer research suggests that not only do the 3D models provide a realistic platform for studying tumor–immune system interactions, but also open new avenues for the development of more targeted and personalized therapeutic approaches for cSCC. These models allow researchers to recapitulate the histological structure, mutational heterogeneity, and cellular complexity of human tumors, making them valuable tools for preclinical drug screening and predicting patient-specific responses. However, despite their promise, 3D skin models still face important limitations. They are often costly, technically demanding, and suffer from limited reproducibility. Furthermore, while paracrine signaling can be mimicked (e.g., through the use of conditioned media), this approach cannot fully replicate the dynamic, multicellular crosstalk that occurs in vivo. In contrast, animal models—particularly murine models—offer a more complete biological context, including systemic immune responses, vascularization, and the contribution of multiple organ systems. That said, murine models also present notable drawbacks, such as species-specific differences in skin structure, immune function, and tumor behavior, which can limit the translational relevance of findings to humans. Therefore, while 3D in vitro models are invaluable for dissecting specific molecular and cellular mechanisms and offer greater control over experimental variables, animal models remain essential for capturing the full complexity of tumor biology. A combined approach, leveraging the strengths of both systems, will likely yield the most informative and clinically relevant insights into cSCC pathogenesis and therapy. Recapitulating the histological, mutational heterogeneity, and the complexity of cell interactions within human cancers is fundamental for preclinical drug screening and for predicting patient outcomes.

## Figures and Tables

**Table 1:** Immune TME regulators in cSCC.

NAME	TYPE	PHYSIOLOGICAL ROLE	ROLE IN CSCC
CD200	Receptor	Immunomodulator [88]	Inhibits DC maturation; enhances proteinases production [88,89]
SHCBP1	Signaling regulator	T cell development [90]	Reduces CD8+ T cells; Increases TAM [91]
PD-L1 AND -L2	Receptor	Modulates T cell activity [92]	Supports immune evasion [93]
CTLA-4	Receptor	Regulates T cell activation [94]	Suppresses immune Response [41]
LAG-3	Receptor	Regulates T cell activation [95]	Downregulates T cell activity [41]
IL-6	Receptor	Proinflammatory	Overexpression promotes tumor growth [96]
IL-24	Cytokine	Anti-inflammatory	Increases MMP-7 [97]
TGF-Β	Cytokine	Anti-inflammatory	Suppresses antitumor immunity [98]
IL-22	Cytokine	Anti-inflammatory	Reduces IFN-γ production by Th1 cells [99]
IL-27	Cytokine	Modulates inflammation [100]	Favors Th1-like TME [101]
VEGF	Angiogenic factor	Regulates formation of blood and lymphatic vessels	Overexpression promotes tumor growth [102]
S100A8 AND A9	Alarmins	Activates inflammatory response after skin injury	Chronically stimulates inflammatory cells’ infiltration; stimulate secretion of MMPs [103]
MMPS	Proteases	Models ECM	Favors tumor expansion [104]
SERPIN A1, B3	Protease inhibitor	Models ECM	Inhibits apoptosis; stimulates proliferation [105]
OPN	Protease	Models ECM	Enhances metastasis by promoting cells migration [106]

## Data Availability

No new data were created or analyzed in this study.
